# Development, implementation and evaluation of a sustainable medication-use process in the intensive care unit: a lean-based quality improvement study

**DOI:** 10.1007/s11096-026-02214-z

**Published:** 2026-10-20

**Authors:** Rou Qing Chen, Shirley Xie, Tim Frenzel, Sanne Percic-van Doorne, Bart van den Bemt, Stephanie Natsch, Charlotte Bekker

**Affiliations:** 1 Department of Pharmacy, Pharmacology and Toxicology, Radboud Institute for Health Sciences, Radboud University Medical Centre, Nijmegen, The Netherlands; 2 Department of Intensive Care, Radboud Institute for Health Sciences, Radboud University Medical Centre, Nijmegen, The Netherlands; 3 Department of Pharmacy, Sint Maartenskliniek, Nijmegen, The Netherlands

**Keywords:** Intensive care units, Medical waste disposal, Medication systems, hospital, Sustainable development, Total quality management

## Abstract

**Background:**

In the Intensive Care Unit (ICU), substantial quantities of medication are wasted, negatively affecting ecological and economic sustainability.

**Aim:**

This study aimed to develop, implement, and evaluate an ecologically and economically sustainable medication-use process to reduce medication waste in the ICU of a Dutch academic hospital.

**Method:**

This lean-based quality improvement study comprised three phases: (I) Three-week baseline medication waste assessment in terms of quantity (i.e., disposed medication units) and economic value per 100 ICU beds per day. (II) Development and implementation of a sustainable medication-use process using lean principles. Causes of medication waste were identified among hospital staff using qualitative methods, and targeted interventions were identified and implemented accordingly. (III) Post-implementation effect evaluation of the sustainable medication-use process on medication waste.

**Results:**

At baseline, a mean total of 113 units, worth €1037, were wasted daily per 100 ICU beds. Causes for medication waste were: insufficient knowledge among hospital staff about quantities and impacts of medication waste; suboptimal medication ordering, supplying and preparing, resulting in unnecessary medication waste; and patient’s therapy changes/discontinuation. Interventions implemented were: awareness campaigns on medication waste; adjustment of protocols and work processes to prevent excess order, supply and preparation of medication; and dispensing individually labelled medication to the patient following transfer or discharge. Implementing these interventions reduced medication waste by 31.8% to 77 units per 100 ICU beds per day, and economic losses decreased by 18.4% to €846.

**Conclusion:**

Implementation of a sustainable medication-use process targeting staff’s knowledge and work processes, reduced the extent of wasted medications in the ICU and associated costs.

**Supplementary Information:**

The online version contains supplementary material available at https://doi.org/10.1007/s11096-026-02214-z.

## Impact statements

- Medication waste on hospital wards negatively impacts resource efficiency and contributes to avoidable environmental burden and associated costs.
- Lean processes could be used to reduce medication waste in the ICU, thereby improving sustainability of healthcare.
- Pharmacy and ICU staff can play a key role in sustainability stewardship through more collaboration across medication prescribing, preparation, distribution, and disposal processes.

## Introduction

Healthcare is facing sustainability challenges as climate change adversely affects human health, while healthcare itself contributes to these changes [1]. Annually, the sector accounts for nearly 5% of global greenhouse gas emissions [1], and approximately 7% of national emissions in the Netherlands [2]. Medications contribute to around 20–40% of healthcare-related emissions [3, 4]. Environmental pollution from medications occurs from production and usage to incineration of unused medications [3, 5]. Medication that remains unused throughout the medication supply chain is considered medication waste [6]. Disposal thereof is especially wasteful, as it affects both environmental and economic sustainability [7, 8].

To reduce the environmental and economic impact of unused medications, a shift towards sustainable medication use is needed. The key for this transition is to identify causes of medication waste and targets for potential interventions. Within hospitals, medication waste arises from various causes, e.g., inadequate stock management, inefficient medication ordering and delivery procedures, or poor usability of available Information Technology (IT) systems [9]. At the patient level, therapy changes, treatment discontinuation, and medication substitution upon hospital admission can also contribute to medication waste [9, 10].

In intensive care units (ICUs), patients frequently require multiple medications and experience frequent treatment modifications. A cross-sectional study found that over 40% of unused medications in a tertiary care hospital originated from the ICU, with approximately 70% requiring disposal [11]. Another observational multicentre study showed 38% of prepared medications in ICUs and operating rooms remained fully unused and were discarded [12]. Clearly, the ICU is at risk of medication waste, requiring process optimisation to become more sustainable.

Lean management could be used as a guide to streamline the medication-use process. Lean management is a systematic quality improvement methodology which optimises efficiency by minimising waste and maximising value for participants involved [13]. In healthcare, it has been widely adopted and empirical evidence shows lean interventions are associated with improved process outcomes, including staff engagement and workflow improvement [14–16]. By focusing on participants’ values and eliminating non-value-added activities, lean principles may aid the design of a streamlined medication-use process that improves efficiency, promotes sustainable medication use, supports adoption into practice, and potentially reduces medication waste [13].

### Aim

This study aimed to develop, implement, and evaluate an ecologically and economically sustainable medication-use process to reduce medication waste in the ICU of a Dutch academic hospital.

## Method

### Study design and setting

A lean-based quality improvement study was conducted in the ICU and pharmacy departments of the Radboud university medical centre (RUMC), the Netherlands, between February 2022 and August 2023. The ICU comprises three units (intermediate care, intensive care, and cardio(thoracic) care) with 48 available beds. The intermediate care unit treats patients requiring a level of care between the general ward and intensive care. The intensive and cardio(thoracic) care units provide the highest level of patient care, including full organ support with mechanical ventilation and renal replacement therapy. The cardio(thoracic) care unit primarily treats patients following cardiothoracic surgery and those requiring extracorporeal membrane oxygenation. Each unit has a medication room containing floor stock and patient-labelled medications. Floor stock medications are supplied as whole original packages and patient-labelled medications are supplied as whole packs or as five-unit doses when unit-dose packaging is available. Inventory levels are monitored electronically and pharmacy staff replenish floor stock whenever minimum quantities are reached. Patient-labelled medication are dispensed by the pharmacy upon request and may be returned to the pharmacy if predefined criteria are met, i.e., unopened, undamaged, correctly stored, minimum economic value of €25, and remaining shelf life ≥6 months. A medication traceability system links each medication unit to patients, operators, and preparation steps [17]. Most parenteral medications are prepared by ICU nurses but certain high-risk medications (e.g., intravenous chemotherapy, biologic agents) are prepared by pharmacy staff.

Lean management guided the development and implementation of a sustainable medication-use process, applying four principles: identifying value, mapping the value stream, creating flow, and establishing pull [18]. The fifth principle, seeking perfection, was not applied because this study evaluated implemented interventions rather than continuous improvement. The medication-use process was defined as the continuum of prescribing, preparation, dispensing, administration, adherence, and monitoring of medication use, including the behaviours and systems influencing these stages.

### Data collection

This study consisted of three phases: (I) problem assessment, (II) development and implementation of a sustainable medication-use process, and (III) process evaluation.

#### Phase I: Problem assessment

Medication waste was measured during a three-week period in June 2022. ICU staff disposed of all unused medication in designated waste bins in medication rooms following usual practice. The following information of disposed medication was registered: name, dosage, dosage form (e.g., tablet, syringe), quantity disposed (e.g., number of tablets, volume of syringes), and expiration date. For liquid medications the volume was estimated, with a residual volume of ≥8% of the original volume classified as waste.

Mean bed occupancy and patient demographics were extracted from the hospital information system to compare pre- and post-implementation periods. The following patient demographics data were recorded: sex, age at admission, duration of ICU admission, admission type (planned/unplanned), whether the patient died during ICU admission, Acute Physiology and Chronic Health Evaluation IV (APACHE IV) score, and ventilation days.

#### Phase II: Development and implementation of a sustainable medication-use process

***Step 1: Identifying value***

Perspectives on medication waste were assessed through individual semi-structured interviews with staff engaged in the medication-use process (two ICU nurses, two medical specialists, two pharmacy technicians, two pharmacists, and one logistic employee responsible for medication inventory). These interviews were conducted using a predefined interview guide (see Supplementary material), developed within the research team comprising three clinicians, one senior researcher, one junior researcher, and two students. Questions included were whether they recognise medication waste, are aware of its extent, how frequently they encounter it, and what their perceptions are regarding its reduction. All interviews were performed in person (mean duration of 30 min per person), audio recorded, transcribed verbatim, and preceded by verbal informed consent.

***Step 2: Mapping the value stream***

A researcher observed the entire medication-use process in the ICU and pharmacy, recorded each step undertaken, and highlighted steps potentially contributing to medication waste, ultimately creating a value stream map of the medication-use process. These potential steps were coupled to the identified causes for medication waste derived from the interviews and focus groups depicted in step 3.

***Step 3: Creating flow and establishing pull***

Semi-structured individual interviews and focus groups with ICU and pharmacy staff were conducted to identify causes for medication waste and targets for medication waste-minimising interventions. Interviews from Steps 1 and 3 formed a single data collection phase. The interview guide covered both the identification of perspectives regarding medication waste, i.e., identifying value, and perceived causes of medication waste, i.e., creating flow.

Firstly, individual interviews explored participants’ perceived causes of medication waste within the medication-use process (i.e., prescribing, preparing, ordering, delivery, and return processes; IT systems; and communication between ICU and pharmacy staff). Subsequently, focus groups elaborated on these identified causes to gain a more in-depth understanding. Interview participants were invited to participate in the focus groups to allow for iterative data collection and deeper reflection on previously identified themes. The first two focus groups each included ICU nurses, and addressed their work processes and behaviour, whether this could contribute to medication waste, and potential solutions for improvement. The third focus group included two ICU nurses, two pharmacy technicians, and one logistic employee, and was designed to stimulate discussion and interaction between both departments, and addressed causes and potential solutions within the logistic process (i.e., medication ordering, delivery, and return). All interviews and focus groups were recorded and transcribed verbatim. Guides are provided in the Supplementary material.

Based on the identified causes and the mapped medication-use process, a multidisciplinary working group, consisting of two researchers, one hospital pharmacist, one ICU nurse, and one medical specialist, developed interventions to optimize the medication-use process. Proposed interventions per identified cause were discussed until consensus was reached. Following the pull principle, interventions were designed to streamline the medication-use process by addressing immediate user needs [18].

***Step 4: Implementing the medication waste-minimising interventions***

The defined interventions were implemented through various activities over a six-months period, with some interventions implemented concurrently and others sequentially. These included protocol and workflow changes, departmental meetings, a sustainable medication week, and distribution of factsheets and newsletters addressing medication waste. During the sustainable medication week, results of the problem assessment and proposed interventions were presented.

#### Phase III: Process evaluation

Two months after implementation of the final intervention, medication waste was reassessed during a three-week follow-up period in July 2023 following the same procedure as in Phase I. Again, all unused medication was collected and registered, and data regarding patient demographics were extracted.

### Data analysis

#### Statistical analysis

Data were analysed using Statistical Package for the Social Sciences (version 29, IBM Corp., Armonk, NY, USA). Descriptive analysis was used to describe population demographics, with categorical variables reported as percentages, and numeric variables as means (standard deviation) if normally distributed or medians (interquartile range (IQR)) if non-normally distributed. Statistical differences in patient demographics were analysed using Mann–Whitney U tests for continuous variables and Chi-squared tests for categorical variables. A *p*-value of < 0.05 was considered statistically significant.

The quantity of medication waste was expressed in units, with each unit being the smallest dividable quantity (e.g., one tablet is one unit, one syringe is one unit). The quantity of individually disposed units was adjusted for the bed occupancy and expressed as wasted units per 100 ICU beds per day. The economic value of the units was calculated using pharmacy’s purchase unit prices specific to each assessment period. Pre-post differences were expressed as percentage changes in the rate of wasted units per 100 ICU beds per day.

#### Interviews and focus groups

Data were analysed using ATLAS.ti 24.0.0 for Windows following an inductive coding approach. Initial open coding was performed by one junior female researcher (SX) under supervision of one senior female researcher (CB). Similar open codes were grouped into categories by SX and reviewed by CB. Subsequently, the categories were allocated within the medication-use process. Statements reflecting the identified causes were selected, summarised, and translated into English by SX.

### Ethics approval

The questionnaires and methodology for this study were approved by the accredited Research Ethics Committee at the RUMC (file number: 2025–18093).

## Results

### Patient demographics

A total of 118 patients were admitted to the ICU during the pre-intervention period, with a mean occupation of 33.2 beds per day. During the post-intervention period, a total of 108 patients were admitted, with a mean bed occupation of 34.2 beds per day. No statistically significant differences in patient characteristics were observed between both groups (Table 1). Patient demographic data of patients admitted to the intermediate care unit are missing for both periods due to IT constraints.

**Table 1:** Patient characteristics during the pre- and post-intervention waste assessments ^a^

	Pre-intervention (n=118)	Post-intervention (n=108)
Sex, n (%) Male	80 (67.8%)	76 (70.4%)
Age (years), median (IQR)	64 (55–72)	61 (48–69)
Admission duration (days), median (IQR)	1.1 (0.9–2.7)	1.3 (0.9–3.0)
Planned admission, n (%) Yes	59 (50.0%)	61 (56.5%)
Died during ICU admission, n (%) Yes	14 (11.9%)	15 (13.9%)
APACHE IV score, median (IQR)	65 (53–79)	62 (50–80)
Ventilation days, median (IQR)	1 (0–2)	1 (1–3)

No statistically significance difference was found between the pre- and post-intervention groups. ^a^ Excluding the intermediate care unit

### Value stream mapping and medication waste-minimising interventions

**Fig. 1:**
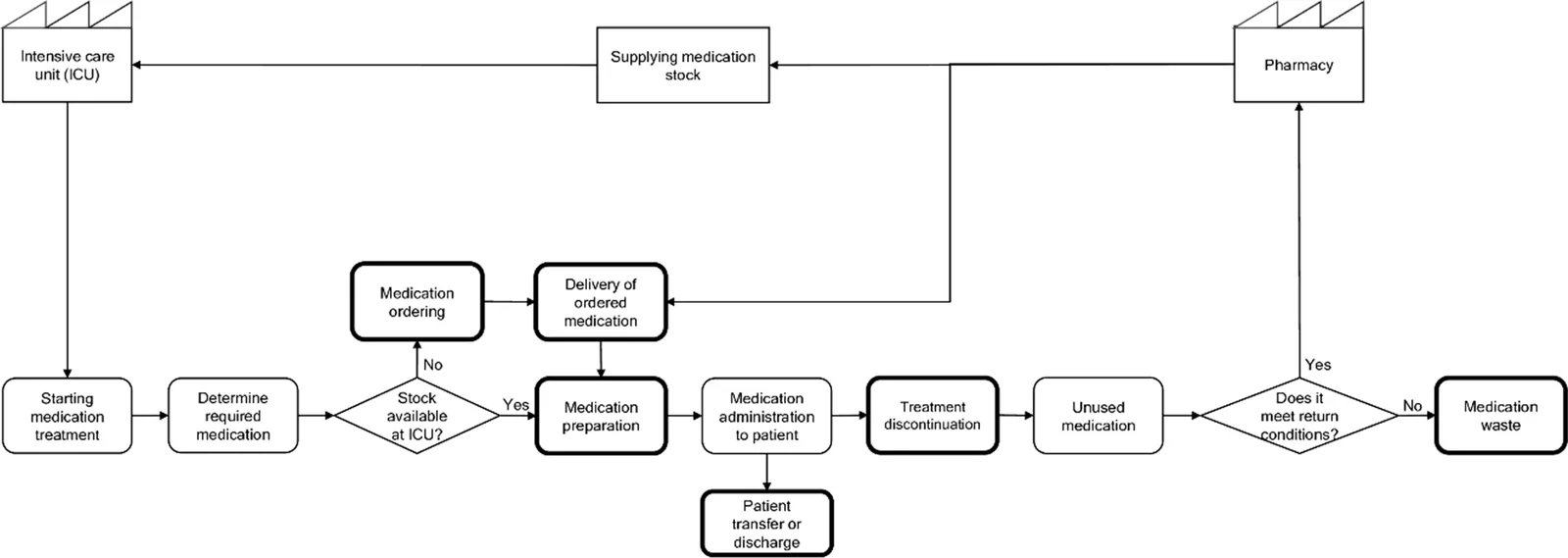
Value stream map of the medication-use process. Framed boxes indicate process steps during which medication waste was likely to occur

Figure 1 illustrates the medication-use process in the ICU and highlights steps contributing to medication waste. ICU and pharmacy staff reported multiple causes of waste across the medication-use process, particularly during medication ordering, delivery, and preparation, as well as following treatment discontinuation and during patient discharge or transfer. These findings informed the development of targeted medication waste-minimising interventions (Table 2). All developed interventions were implemented, except for the revised return policy, which was implemented after the post-intervention period.

**Table 2:** Overview of medication waste process steps, cause of medication waste, and matched waste-minimising interventions

Medication waste in value stream	Cause of medication waste	Waste-minimising intervention
Medication waste	Medication waste is not consistently recognised or acted upon in daily ICU practice due to limited awareness of both the extent of medication waste and its impact, resulting in suboptimal inventory-related behaviour, such as incorrect medication storage, and difficulties in behavioural change.	Increase awareness of medication waste in the ICU through a dedicated sustainable medication week, distribution of factsheets and newsletters addressing medication waste using results of the problem assessment (Phase I).
Medication ordering	Pharmacy will deliver a 5-day stock of every medication if no order quantity is specified, which may result in excess stock.	Establish clear medication ordering working agreements. These include mandatory completion of ordering quantities via the hospital information system by nurses and physicians, and restricting pharmacy orders to a maximum of 3-day stock per request.
Delivery of ordered medication	Pharmacy-delivered medication may be misplaced or lost due to the lack of a delivery point in the ICU, resulting in the re-ordering and excess stock.	Set a standard delivery point in the ICU in the medication room of each main unit.
Medication preparation	ICU nurses prepare parenteral medication in advance and in quantities exceeding immediate demand to prevent potential stock shortages, leading to unused doses when changes in treatment regimens occur or when prepared medications reach their expiration date.	Use ready-to-administer syringes instead of preparing syringes whenever possible.
		Use a standardised protocol for medication preparation in the ICU. This includes preparing medications no more than 2 h in advance; communicating which medication is prepared during handover-moments; storing patient-labelled prepared medication in cupboards organised by room number; and preferring bolus administration over continuous infusion where clinically appropriate.
		Review current shelf-life of intravenous medication in accordance with the Dutch Good Manufacturing Practices hospital pharmacy (GMP-Z3). This includes extending the expiry time upon administration of ready-to-administer syringes and basic infusion fluids without active medication (e.g., sodium chloride 0.9%, glucose 5%) where appropriate; updating the local parenteral medication handbook; and extending the expiry dates of prepared parenteral medication where feasible (while complying to guidelines).
Treatment discontinuation & Patient transfer or discharge	Unused medication is discarded in the ICU rather than returned to the pharmacy because of strict return criteria and the labour-intensive nature of the return process for ICU staff.	Revise the medication return policies to facilitate returns, including removing the minimum economic value threshold and shortening remaining shelf-life requirements to ≥3 months; and introducing dedicated return bins for ICU staff to deposit return medication, which are collected weekly, rather than requiring ICU staff to bring the medication to the pharmacy themselves. ^a^
Patient transfer or discharge	Patient-labelled medication cannot be reused for another patient and is discarded when remaining unused in the ICU.	Dispense patient-labelled medication to the patient upon discharge or transfer to a different hospital ward.

^a^This intervention was implemented after the post-intervention waste assessment

### Medication waste pre- and post-intervention

During the pre-intervention waste assessment, 113 medication units per 100 ICU beds per day were wasted (Table 3), with an economic value of €1037 per 100 ICU beds per day (Table 4). The majority of medication waste consisted of oral medications (30.1 units per 100 beds per day), ampoules and vials (29.8 units per 100 beds per day), and prepared syringes (24.9 units per 100 beds per day) (Table 3). Prepared syringes primarily contained medication from the following therapeutic subgroups: general anaesthetics, antipsychotics, cardiac stimulants, and analgesics. Approximately 69% of all disposed syringes (including ready-to-administer syringes) contained at least 50% of their original volume, and 38% were completely unused at the time of disposal.

**Table 3:** Wasted units across the ICUs during the medication waste assessment periods, including pre- and post-intervention differences

Type of medication	Units of waste per 100 ICU beds per day		Difference pre-post
	Pre-intervention	Post-intervention	
Ampoules and vials	29.8	2.3	- 92.4%
Infusion bags	11.6	8.1	- 30.1%
Ready-to-administer syringes	13.9	17.1	+ 23.3%
Prepared syringes	24.9	29.1	+ 16.8%
Oral medication (including tablets, capsules, sachets)	30.1	20.3	- 32.7%
Others ^a^	2.7	0.1	- 94.7%

Total units of waste	113.0	77.0	- 31.8%

^a^ Including pens, nasal sprays, dermal preparations, and transdermal patches

**Table 4:** Economic value of wasted units during the waste assessment periods, including pre- and post-intervention differences

Type of medication	Economic value (€) of waste per 100 ICU beds per day per ICU		Difference pre-post
	Pre-intervention	Post-intervention	
Ampoules and vials	387.0	22.9	- 94.1%
Infusion bags	23.6	3.1	- 87.0%
Ready-to-administer syringes	139.1	159.6	+ 14.8%
Prepared syringes	357.4	656.5	+ 83.7%
Oral medication (including tablets, capsules, sachets)	33.3	4.0	- 87.9%
Others^a^	96.9	0.1	- 99.9%

Total units of waste	1037.3	846.2	- 18.4%

^a^Including pens, nasal sprays, dermal preparations, and transdermal patches

After the implementation of the interventions, 77.0 medication units per 100 ICU beds per day were wasted (Table 3), with an economic value of €846 per 100 ICU beds per day (Table 4). An overall reduction of 31.8% in wasted units per 100 ICU beds per day was achieved. Although most types of medication waste were reduced during the post-intervention period, there was an observed increase in the disposal of ready-to-administer syringes and prepared syringes.

The quantities and types of medication wasted varied among the different ICUs (Table 5). While a waste reduction was observed in the intensive care and cardio(thoracic) care units, a 77% increase was observed in the intermediate care unit. Additional results regarding the specific types of medication can be found in the Supplementary material.

**Table 5:** Wasted units per type of medication per ICU during the waste assessment periods

Type of medication	Units of waste per 100 ICU beds per day					
	Intermediate care		Intensive care (excluding cardio)		Cardio(thoracic) care	
	Pre	Post	Pre	Post	Pre	Post
Ampoules and vials	1.0	0.0	43.0	0.7	31.0	5.0
Infusion bags	8.3	23.1	2.5	7.7	22.4	1.1
Ready-to-administer syringes	8.3	12.1	16.6	30.0	14.1	6.8
Prepared syringes	13.0	13.3	21.0	50.2	34.7	15.8
Oral medication (including tablets, capsules, sachets)	10.4	24.9	59.6	26.2	10.5	12.0
Others^a^	0.5	0.0	6.1	0.4	0.3	0.0

Total units of waste	41.5	73.4	148.7	115.1	113.0	40.8

^a^Including pens, nasal sprays, dermal preparations, and transdermal patches

## Discussion

This lean-based quality improvement study demonstrated that a sustainable medication-use process in the ICU and pharmacy departments resulted in a one-third overall medication waste reduction and 20% cost savings.

The overall reduction in medication waste shows the implemented interventions were most likely effective. While the overall effect was observed among most ICUs, paradoxically, an increase in wasted medication units was seen in the intermediate care unit. This may relate to differences in patient populations between the pre- and post-intervention periods –as patient demographics were unavailable for this unit–; to staff’s adherence to the implemented interventions; or to incidental large disposals of sodium chloride infusion bags post-intervention. The most frequently wasted medication types included general anaesthetics, cardiac stimulants, and analgesics, comparable with existing literature [12, 19], reflecting the common use of continuous sedation, vasopressors and pain management medications in ICU practices. During the pre-intervention period, 38% of all disposed syringes were completely unused, consistent with findings from previous studies [12, 20]. Post-intervention, reductions were observed in wasted ampoules, vials, and infusion bags, alongside an increase in ready-to-administer syringes. The latter reflects the result of one of the implemented interventions. Favouring the use of ready-to-administer syringes offers sustainability benefits, including eliminating multi-dose ampoules and vials use, related production and handling procedures, reducing waste from leftover product, and a longer shelf-life [21, 22]. Although unit prices of these syringes are generally higher, their contribution to the overall economic value of medication waste remained limited in this study. Furthermore, reductions in other dosage forms may reduce resource use, including energy and water consumption, and associated emissions [23, 24].

As global healthcare-related greenhouse gas emissions continue to rise [1], embedding sustainability into everyday clinical practice is becoming increasingly urgent [25]. This study demonstrated a sustainable medication-use process is successful in reducing medication waste in the ICU. Importantly, not all proposed interventions were implemented during the study period, suggesting that further reductions are feasible. Despite the observed reduction, medication waste remained present. While not all waste is preventable, research suggests that up to 80% of medication waste in the ICU is potentially avoidable [12], which demands more initiatives to be implemented. Given the significant and rising financial and environmental implications associated with unused medications [3, 26] −particularly in high-risk departments such as ICUs [27]− and the risk of medication shortages [19], process optimisation is essential. Interventions targeting both staff’s perspectives and underlying process inefficiencies may support more effective and sustainable medication management [28], while ensuring availability of critical drugs. In addition, close collaboration between ICU and pharmacy staff may enhance sustainability by improving communication, optimising medication-related processes, and facilitating implementation of practice changes across disciplines [29]. Furthermore, health authorities and hospital administrators should prioritise medication waste reduction by establishing standardised waste-monitoring protocols to ensure continuous evaluation and optimisation of interventions, ultimately fostering a sustainable medication management system.

Moreover, to achieve broader sustainability, interventions should also address other domains such as medical waste management and energy consumption [30, 31]. A lack of knowledge and awareness, as seen in this and other studies [19, 32, 33], as well as a lack of responsibility [34], are widely recognised as key barriers to change. Structured education, awareness campaigns, and local multidisciplinary teams may support behavioural changes and implementation of sustainability practices [32–36]. Organisational and professional leadership may also contribute in advancing these efforts [32]. Ultimately, such actions will contribute to a more sustainable ICU, safeguarding both patient care and the planet for future generations.

### Limitations

This study has several limitations. First, the economic value of wasted medications was calculated using pharmacy’s list prices, which differ from publicly known prices due to confidential agreements [37]. Consequently, the actual cost savings presented in this study may differ in other hospitals or countries. The reasons for medication disposal were not collected and therefore any incidental large disposal of certain types of medication cannot be explained. Additionally, multiple interventions were implemented simultaneously, making it challenging to estimate the effect of each single intervention undertaken. However, the combined implementation of the developed interventions in this study has been shown to be effective in reducing medication waste. This study was not designed to detect statistical differences in intervention effectiveness in reducing medication waste, because this study focused on the development of the sustainable medication-use process, and a relatively short evaluation period was used. Furthermore, patient characteristics for the intermediate care unit were unavailable due to IT difficulties, limiting the assessment of potential confounding. Moreover, this study did not evaluate potential unintended impacts such as staff workload or patient safety outcomes, which may influence the broader impact of the adapted medication-use process. In addition, data collection was limited to June and July, and seasonal variations in ICU admissions and medication use may affect generalisability. Lastly, the interventions used in this study were tailored to the local context and may require adaptation for other settings.

## Conclusion

Carefully reviewing the medication-use process together with involved hospital staff combined with a waste assessment could help to establish a more sustainable medication-use process and could reduce medication waste. Potential approaches include enhanced staff training, awareness initiatives, optimisation of inventory management, and revision of (preparation) protocols. Further research may examine the effectiveness of individual interventions, associated cost savings, and scalability across other healthcare settings.

## Supplementary Information

Below is the link to the electronic supplementary material.

Supplementary file1 (PDF 230 KB)

## Data Availability

The first and the last authors have full access to all the data in the study and take responsibility for the integrity of the data and the accuracy of the data analysis. The data that support the findings of this study are available from the corresponding author upon reasonable request.
