# Supplementary material for: Development, implementation and evaluation of a sustainable medication-use process in the intensive care unit: a lean-based quality improvement study
Source: Int J Clin Pharm. 2026 Oct 20;48(6):2373–82. doi: 10.1007/s11096-026-02214-z (PMC13731704; doi:10.1007/s11096-026-02214-z)
Supplement: Supplementary file 1 [file 11096_2026_2214_MOESM1_ESM.pdf]

Development, implementation and evaluation of a sustainable medication process in the intensive care unit: a lean-based quality improvement study

Supplementary material

International Journal of Clinical Pharmacy

Rou Qing Chen, Shirley Xie, Tim Frenzel, Sanne Percic-van Doorne, Bart J.F. van den Bemt, Stephanie Natsch, Charlotte L. Bekker*

*Department of Pharmacy, Pharmacology and Toxicology, Radboud Institute for Health Sciences, Radboud University Medical Center, Nijmegen, the Netherlands.
Charlotte.Bekker@radboudumc.nl

Contents

Interview guide – Individual interviews 2
Topic guide – Focus groups 1 and 2 4
Topic guide – Focus group 3 6
Additional results 8

Interview guide – Individual interviews

*[Introduction of interviewers] *names*,* students who are conducting research into medication waste, specifically how this occurs in the intensive care unit (ICU) and the pharmacy department.

Thank you for agreeing to participate in this study. The aim of this study is to identify factors that contribute to medication waste in the ICU. This is done by interviewing pharmacists, ICU physicians, ICU nurses, and pharmacy (logistics) employees. During the interview, questions will be asked about the prescription, use, and storage of medication and medication waste. The interview will take approximately 30 minutes.

With your consent, the interview will be audio recorded. This recording will allow us to fully process the data at a later time, ensuring that no valuable information is lost. The content of this interview will be used exclusively for this study. It will be processed anonymously and stored on a secure system and only researchers will have access to it. The recording will be deleted once the study has been completed and transcription is completed.

*[IF: <u>verbal consent</u> was obtained] *Turn on sound recording**

Definition of the problem

The following definition of medicine wastage is given: "Any medication that remains unused, passed its expiry date, or is partially unused."

- Do you recognize medication waste in your department?
- Do you keep track on the amount of medication waste? Why or why not?
- Do you ever encounter medication waste during your work activities? When and how often?
- What do you think are the consequences of medication waste?
- To what extent do you think it is important to reduce medication waste? And why?
- To what extent is there awareness in the department of the consequences of medication waste?

We would like to use the following questions to identify the possible causes of medication waste.

Causes of medication waste

- How do you think medication waste occurs in this department?
- In which daily activities do you personally deal with medication?
- How do you think medication waste occurs in your personal work activities?

ICU and pharmacy

- How are medications prescribed?
- To what extent is medication waste taken into account in the prescribing process? Why/why not?
- How is the quantity of medication determined when ordering medication? Who is responsible for this?
- To what extent is medication waste taken into account in the ordering process? Why/why not?
- How are medication prepared for administration and who is responsible for this?
- To what extent is medication waste taken into account when preparing medication? Why/why not?

ICT-systems

- What ICT software is used to prescribe/order/dispense medication in the department?
- How are the quantities determined?
- To what extent is medication waste taken into account in the ICT systems used by the department? Why/why not?

Logistical processes

- How is the quantity of medication to be purchased/ordered determined? Is this done automatically or do you play a role in this yourself?
- To what extent is medication waste taken into account in the purchasing methods? Why/why not?
- How many medication are in stock in this department? How is this stock determined and monitored?

Returned medication

- What do you do with unused medication?
- How is the return of unused medication organized?
- Which medication cannot be returned and what happens to them?
- Are unused medication exchanged between different departments? And why is this the case?

Internal communication

- How is cooperation between the ICU and the pharmacy organized with regard to medication?
- To what extent is medication waste taken into account in communication within the department and between different departments? Why/why not?

Other

- What are the differences in the processes for medication “in stock” and “patient-labelled”?
- And for oral versus intravenous medication?

We have asked all our questions. Do you have any suggestions or ideas of your own, or would you like to add anything?

Topic guide – Focus groups 1 and 2

Reason
Based on previous collected data (individual interviews), there are a number of themes that require further investigation before solutions can be formulated and implemented.

Objective
To investigate factors in the medication process and in the behavior of employees of the intensive care unit and pharmacy department that contribute to medication waste.

Design
Focus groups (4-6 people per session) with nurses discussing both causes and solutions.

Participants
Nursing staff from the ICU department (not pediatric ICU).

Methods
Each session will start with a short presentation (max. 10 minutes) about the purpose of the meeting, the rules, and the results of the waste measurement and individual interviews. Afterwards, the discussion will begin.

- Themes are shown in *italics*;
- Main questions are indicated with numbers;
- Suggestion for further exploration are indicated with a ○ symbol.

1. What is your view on the measured medication waste (extent and type of medication [66% pre-filled syringes])?
2. To what extent are you aware of this (*awareness*)?

We would like to hear your opinion on which factors contribute to the waste of pre-filled syringes, so that we can come up with appropriate solutions.

Causes

3. What are possible reasons why medication is prepared but afterwards not (fully) administered and ends up in the waste bin?
    - o Working with fixed protocols; fixed quantities/syringe sizes
    - o Timing of preparation during a shift
    - o Help from colleagues
    - o Order changes
4. Do you ever prepare medication (syringes) in advance? If so, how far in advance (next administration time, entire shift, preparation for next shift), in what situations and why (*working ahead*)?
5. Have you ever experienced running out of medication (*anxiety*)? What happened then, how did this affect you? What caused this (*delayed delivery from the pharmacy, lack of time, rush,* etc.)?
6. To what extent do you have insight into medication already prepared by a colleague? How are you informed of this? How do you inform a colleague when you have prepared medication? (*collaboration, communication, habits, expectations*)
    - o Is this placed on a fixed location?
    - o Have working agreements been made about this? Are these recorded anywhere?

7. To what extent are you confidence in the current working agreements regarding the preparation of medication (*confidence*)?
    - If you were to rate this on a scale of 1-10, what score would you give? What is needed to increase this score? Why not lower? Why so high? What works well? What does not work well?
8. What other issues do you encounter when preparing medication that could potentially result in waste?

Possible solutions
We have identified a number of causes, namely xx and xx.

9. What could help to address these causes?
    - Depending on the causes, focus on trust, behavior, communication, etc.
    - E.g., how do we ensure a uniform process in which we work in the same way and check medication stock?
    - What do you need from the department, and what from the pharmacy (*provision of information*)?

Topic guide – Focus group 3

Reason
Based on previous collected data (individual interviews), a number of bottlenecks have emerged that require further investigation before solutions can be formulated and implemented.

Objective
To assess current processes related to the ordering and returning of medication by nurses in terms of their impact on medication waste, aimed at developing an improved, more sustainable working method.

Design
Focus groups (4-6 people per session) with pharmacy staff and 1-2 ICU nurses.

Participants
Nursing staff from the ICU department (not pediatric ICU) and staff from the pharmacy department (pharmacist, logistic staff).

Methods
Each session will start with a short presentation (max. 5 minutes) about the purpose of the meeting, the rules, and the results of the waste measurement and individual interviews. Afterwards, the discussion will begin.

- Main questions are indicated with numbers;
- Sub-questions are indicated with letters;
- Suggestion for further exploration are indicated with a ○ symbol.

Ordering method (15 minutes)
Problem: no clear ordering method – unclear who is responsible, form is not completed filled in by everyone (mainly quantities), lack of flexibility in order quantities (only whole packages).

1. Which factors contribute to medication waste within the ordering process?
    a. How does the medication ordering process work in the ICU? (department-based vs. patient-labelled)
        - How do you determine the quantity to order? What do you take into account?
        - Do you check the current stock before ordering?
        - Communication with the prescriber about future policy regarding the medication in question?
        - Filling in order quantities: Do you always do this? Do you find this difficult? Fear of running out?
        - Is the current order form user-friendly?
        - What problems do you encounter when ordering? What could be improved?
    b. What do you check when you receive a (patient-labelled) order?
    c. What agreements are in place regarding the quantity to be delivered?
        - Formal written agreements? Verbal agreements?
        - What do you do if quantities are not specified?
        - Who is responsible for ensuring that the order is correct?
    d. What problems do you encounter in the pharmacy?
        - Where do you think things go wrong?
2. What is needed to optimize this process?

Delivery process (10 minutes)

3. Which factors contribute to medication waste within the delivery process?
    a. Are there any agreements in place regarding the place of delivery of patient-labelled medication?
        - Where is the medication delivered?
        - Is it recorded in the EPD that medication has been ordered for the patient and where it is stored?
    b. Are there any notable issues with the delivery of medication?
        - Is there often sufficient stock, but an order is placed anyway?
    c. What do you think about the current delivery method to the department?
    d. What problems do you encounter in the pharmacy?
4. What is needed to optimize this process?

Departmental stock (10 minutes)

5. How often is the departmental stock counted? By whom? (at least once a year by the departmental manager according to protocols – should this be done more often?)
    - What work instructions/protocols are used for this?
6. What agreements are in place regarding adjustments to departmental stock?
    - How do you determine which stock limits are appropriate?
    - When should adjustments be made downwards? How often?
    - When should adjustments be made upwards? How often?

Return process (15 minutes)

Problem: difficult return process due to strict requirements (part of pharmacy stock, minimum value of 25 euros, minimum shelf life of 6 months, item must be stored correctly and the items/packaging must not be damaged).
<u>*Situation 1*</u>*: Medication is delivered patient-labelled, but is not given to the patient upon discharge. This causes leftover medication, often a full package with only a few tablets missing.*
<u>*Situation 2*</u>*: A nurse has insufficient knowledge about a drug and orders new medication without checking whether it is in stock. After the order has been placed, the desired medication is found, but the order has already been placed.*

7. Which factors contribute to medication waste within the return process?
    a. What other situations besides those mentioned above occur? And why?
        - Is medication from patients' home being continued during hospitalization?
        - Why is medication not given to patients upon discharge?
        - How can we prevent unnecessary orders from being placed?
    b. According to which standards/guidelines have the current requirements for returns been drawn up?
        - Are these still up to date?
        - Can this be reviewed?
    c. What do you think is the problem with the medication return process?
        - Is filling in the return form seen as burdensome?
        - Is processing the return form seen as burdensome?
        - Are the conditions for returning medication too strict?
8. What options are there for optimizing this process?
    - Adjust the threshold value? Accept opened boxes as well? Reduce the shelf life? What to do with non-pharmacy stock?

Additional results

Table S1. Top 5 medications with the highest wasted quantities pre- and post-implementation.

**Total (mean over all ICUs)**				
	**Pre-implementation**		**Post-implementation**	
	**Medication name**	**Units of waste per 100 ICU beds per day**	**Medication name**	**Units of waste per 100 ICU beds per day**
**1**	Dornase alfa 1mg/ml nebulizer fluid ampule 2ml	7.6	Norepinephrine 0.1mg/ml RTA-syringe	7.6
**2**	Nortriptyline 25mg tablet	7.5	Propofol 20mg/ml prepared syringe	6.6
**3**	Norepinephrine 0.1mg/ml RTA-syringe	7.3	Hydroquinine 100mg tablet	5.5
**4**	Sodium chloride 0.9% infusion bag	6.3	Sodium chloride 0.9% infusion bag	3.7
**5**	Aztreonam 75mg powder and solvent for nebulizer solution	4.6	Insulin aspart 1IE/ml RTA-syringe	3.3

**Intermediate care**				
	**Pre-implementation**		**Post-implementation**	
	**Medication name**	**Units of waste per 100 ICU beds per day**	**Medication name**	**Units of waste per 100 ICU beds per day**
**1**	Sodium chloride 0.9% infusion bag	5.2	Sodium chloride 0.9% infusion bag	12.7
**2**	Norepinephrine 0.1mg/ml RTA-syringe	5.2	Norepinephrine 0.1mg/ml RTA-syringe	6.4
**3**	Nimodipine 30mg tablet	3.1	Pramipexole 0.088mg tablet	6.4
**4**	Paracetamol 500mg tablet	2.1	Paracetamol 500mg tablet	5.8
**5**	Dexmedetomidine 10mcg/ml in glucose 5% prepared syringe	1.6	Pheneticillin 250mg capsule	5.2

**Intensive care**				
	**Pre-implementation**		**Post-implementation**	
	**Medication name**	**Units of waste per 100 ICU beds per day**	**Medication name**	**Units of waste per 100 ICU beds per day**
**1**	Nortriptyline 25mg tablet	18.8	Hydroquinine 100mg tablet	13.7
**2**	Baclofen 25mg tablet	9.6	Norepinephrine 0.1mg/ml RTA-syringe	12.6
**3**	Ofloxacin 3mg/ml eyedrops 0.5ml	8.6	Propofol 20mg/ml prepared syringe	12.5
**4**	Apixaban 5mg tablet	8.3	Sufentanil 5mcg/ml in sodium chloride 0.9% RTA or prepared syringe	6.3
**5**	Norepinephrine 0.1mg/ml RTA-syringe	6.4	Remifentanil 0.05mg/ml in sodium chloride 0.9% prepared syringe	4.6

Cardio(thoracic) care				
	**Pre-implementation**		**Post-implementation**	
	**Medication name**	**Units of waste per 100 ICU beds per day**	**Medication name**	**Units of waste per 100 ICU beds per day**
**1**	Dornase alfa 1mg/ml nebulizer fluid ampule 2ml	19.0	Vitamins ampule 10ml	5.0
**2**	Sodium chloride 0.9% infusion bag	11.7	Nimodipine 30mg tablet	4.2
**3**	Aztreonam 75mg powder and solvent for nebulizer solution	11.6	Propofol 20mg/ml prepared syringe	4.1
**4**	Norepinephrine 0.1mg/ml RTA-syringe	9.3	Lorazepam 1mg tablet	3.3
**5**	Propofol 20mg/ml prepared syringe	5.0	Norepinephrine 0.1mg/ml RTA-syringe	3.3

Table S2. Top 5 medications with the highest economic value of waste pre- and post-implementation.

**Total (mean over all ICUs)**				
	**Pre-implementation**		**Post-implementation**	
	**Medication name**	**Economic value (€) of waste per 100 ICU beds per day per ICU**	**Medication name**	**Economic value (€) of waste per 100 ICU beds per day per ICU**
**1**	Dornase alfa 1mg/ml nebulizer fluid ampule 2ml	150.0	Argipressin 1IE/ml prepared syringe	145.7
**2**	Aztreonam 75mg powder and solvent for nebulizer solution	132.9	Propofol 20mg/ml prepared syringe	143.2
**3**	Propofol 20mg/ml prepared syringe	98.1	Alteplase 0.1mg/ml in sodium chloride 0.9% prepared syringe	77.1
**4**	Posaconazole 40mg/ml oral suspension	76.44	Insulin aspart 1IE/ml RTA-syringe	53.7
**5**	Milrinone 0.4mg/ml in glucose 5% prepared syringe	70.9	Norepinephrine 0.1mg/ml RTA-syringe	52.4

**Intermediate care**				
	**Pre-implementation**		**Post-implementation**	
	**Medication name**	**Economic value (€) of waste per 100 ICU beds per day per ICU**	**Medication name**	**Economic value (€) of waste per 100 ICU beds per day per ICU**
**1**	Neostigmine 0.1mg/ml in sodium chloride 0.9% prepared syringe	50.7	Alteplase 0.1mg/ml in sodium chloride 0.9% prepared syringe	385.5
**2**	Norepinephrine 0.1mg/ml RTA-syringe	44.3	Insulin aspart 1IE/ml RTA-syringe	96.3
**3**	Piritramide 10mg/ml prepared syringe	35.2	Nicardipine 5mg/ml prepared syringe	60.8
**4**	Labetalol 5mg/ml prepared syringe	28.3	Labetalol 5mg/ml prepared syringe	50.7
**5**	Nicardipine 0.5mg/ml in sodium chloride 0.9% prepared syringe	27.3	Norepinephrine 0.1mg/ml RTA-syringe	43.7

Intensive care				
	**Pre-implementation**		**Post-implementation**	
	**Medication name**	**Economic value (€) of waste per 100 ICU beds per day per ICU**	**Medication name**	**Economic value (€) of waste per 100 ICU beds per day per ICU**
**1**	Posaconazole 40mg/ml oral suspension	191.0	Argipressin 1IE/ml prepared syringe	364.1
**2**	Anakinra 100mg/0.67ml RTA-syringe	158.6	Propofol 20mg/ml prepared syringe	275.7
**3**	Propofol 20mg/ml prepared syringe	151.0	Norepinephrine 0.1mg/ml RTA-syringe	87.6
**4**	Amphotericin B/colistin/tobramycin 200/100/40mg suppository	63.7	Rocuronium bromide 10mg/ml prepared syringe	82.7
**5**	Milrinone 0.4mg/ml in glucose 5% prepared syringe	57.9	Nicardipine 0.5mg/ml vial 5ml	80.6

Cardio(thoracic) care				
	**Pre-implementation**		**Post-implementation**	
	**Medication name**	**Economic value (€) of waste per 100 ICU beds per day per ICU**	**Medication name**	**Economic value (€) of waste per 100 ICU beds per day per ICU**
**1**	Dornase alfa 1mg/ml nebulizer fluid ampule 2ml	375.0	Propofol 20mg/ml prepared syringe	82.4
**2**	Aztreonam 75mg powder and solvent for nebulizer solution	332.3	Dexmedetomidine 10mcg/ml in glucose 5% prepared syringe	41.5
**3**	Milrinone 0.4mg/ml in glucose 5% prepared syringe	177.2	Insulin aspart 1IE/ml RTA-syringe	26.6
**4**	Propofol 20mg/ml prepared syringe	94.4	Vitamins ampule 10ml	23.8
**5**	Dexmedetomidine 10mcg/ml in glucose 5% prepared syringe	71.5	Norepinephrine 0.1mg/ml RTA-syringe	21.5
